# Dynamics in the Fitness-Income plane: Brazilian states vs World countries

**DOI:** 10.1371/journal.pone.0197616

**Published:** 2018-06-06

**Authors:** Felipe G. Operti, Emanuele Pugliese, José S. Andrade, Luciano Pietronero, Andrea Gabrielli

**Affiliations:** 1 Departamento de Física, Universidade Federal do Ceará, 60451-970, Fortaleza, Ceará, Brazil; 2 Department of Physics, Sapienza University of Rome, 00185, Rome, Italy; 3 Istituto dei Sistemi Complessi (ISC) - CNR, UoS Sapienza, Dipartimento di Fisica, Università Sapienza, P.le Aldo Moro 5, 00185 - Rome, Italy; 4 International Finance Corporation, World Bank Group, 1818 H Street, 20433 - NW Washington DC - United States of America; University of Warwick, UNITED KINGDOM

## Abstract

In this paper we introduce a novel algorithm, called *Exogenous Fitness*, to calculate the Fitness of subnational entities and we apply it to the states of Brazil. In the last decade, several indices were introduced to measure the competitiveness of countries by looking at the complexity of their export basket. Tacchella *et al* (2012) developed a non-monetary metric called Fitness. In this paper, after an overview about Brazil as a whole and the comparison with the other BRIC countries, we introduce a new methodology based on the Fitness algorithm, called Exogenous Fitness. Combining the results with the Gross Domestic Product *per capita* (GDP_*p*_), we look at the dynamics of the Brazilian states in the Fitness-Income plane. Two regimes are distinguishable: one with high predictability and the other with low predictability, showing a deep analogy with the heterogeneous dynamics of the World countries. Furthermore, we compare the ranking of the Brazilian states according to the Exogenous Fitness with the ranking obtained through two other techniques, namely *Endogenous Fitness* and *Economic Complexity Index*.

## Introduction

Large countries are often characterized by a strong internal heterogeneity between richer regions and poorer hierarchical regions. Just think to the difference between the GDP *per capita* (GDP_*p*_) of the states of New York and Mississippi in the US [1], or the difference between the states of Kerala and Bihar in India [2], or between the unexplored forest of Amazon and the modern state of São Paulo in Brazil [3]. While the recent literature on Economic Complexity focused on countries [4–7], we believe that there are two very strong reasons to extend the scope of the analysis to the subnational level.

The first reason is purely academic. Indeed, sharp differences in economic outcomes in a uniform institutional area—with common cultural background and free movement of workers—are both a theoretical puzzle for traditional economics and an empirical opportunity for the Economic Complexity field. Indeed, the analysis of subnational entities competing on an even playing field is the perfect experimental setup to identify the role of organizational and technical capabilities with respect to more traditional economic factors of analysis. In this paper we will analyze the case of Brazil, to see if the capabilities driven dynamics of a country is replicated at a smaller scale.

The second reason is to improve economic forecasting. Indeed Economic Complexity has been proved to be very effective in forecasting the economic performances of countries [8]. An understanding of subnational entities could give however more accuracy and more detail. It is clear for example that future GDP_*p*_ growth of Brazil will depend not only on further growth of the Southern industrial core, but on the convergence of the other regions. This is crucial both to correctly forecast aggregate Brazil GDP_*p*_ growth and to address the vast internal inequality of the country.

Brazil or, officially, the *Federative Republic of Brazil* is the ninth World economy in the GDP ranking of the year 2015 [9]. Its population is equivalent to 2.81% of the total World population [10] and its large area (8.515.767,049 *km*^2^), divided by its twenty-seven Federative Units [11], make it the fifth largest country of the World [12]. The political and administrative organization of Brazil is hierarchically organized in a sequence of geopolitical structures: Union, states, Federal District and Counties. Each one is autonomous and organized according with the division of powers: legislative, executive, and judiciary. Due to the deep inequalities, but also for the good perspectives of growth, Brazil and the others Latin American countries were often a focus of economic development analysis during the last century [13–17].

Economists usually focus on monetary based indices to analyze economies such as the GDP. However, GDP alone, as shown by different studies [4–7, 18], does not provide deep information about the perspective of growth and development of World countries. Several studies tried to gain information on the unobservable characteristics of countries by looking at stock indices to exploit the “wisdom of the crowd” [19, 20]. In order to gain a direct measure of the country capabilities, the last decade has been marked by a line of research of new indices inspired by the science of complex systems, able to better describe and explain the large scale World economy [4–7, 21–23] and to estimate global and regional inequalities [24, 25].

In this respect, different authors recently introduced two indices: Economy Complexity Index (ECI) [4] and Fitness [5]. Furthermore, Cristelli *et al* [7], through a novel method called *Selective Predictability Scheme* (SPS), showed that the comparison between GDP_*p*_ and Fitness provides a highly performing forecasting tool for several countries.

In this paper, we first present an overview of Brazil as a whole from the point of view of the Economic Complexity approach. In this context we compare its export basket and its Fitness with the ones of the BRIC group of countries (Brazil, Russia, India, and China) [26].

Then, we focus on the comparative study of the economies of the single Brazilian states. Based on the “classical” Fitness algorithm, we introduce a new methodology, called Exogenous Fitness, able to measure the Fitness of subnational entities, and we apply it to the states of Brazil. In analogy with what was proposed in [7], we analyze the coevolution of GDP_*p*_ and Fitness studying in this way the predictability of the economic growth of the Brazilian states.

Furthermore, we compare the Exogenous Fitness with: (i) the (Endogenous) Fitness -i.e, the natural application of the “classical” Fitness algorithm to the subnational entities of a country-; (ii) the results published by the *Dataviva* platform (an application of the ECI algorithm) [27].

The paper is structured as follows: first we introduce the methods and we provide an overview about Brazil. Then, we show the results of the Exogenous Fitness applied to the Brazilian states and the comparisons with the other techniques. Finally, we conclude with a general discussion about the implications of the results with respect to both points of view of scientific community and policy makers. In the Appendix A, we describe in detail the used database.

## Methods

In this section we describe the algorithms and methods involved in the calculation of the states and countries Fitness coupled to the Complexity of exported products.

### Revealed Comparative Advantage (RCA)

The Revealed Comparative Advantage (RCA) [28] is a quantitative criterion to assess the relative advantages of a country, or, in this case, of a Brazilian state, in the export of certain products compared to the average export of those products. Defining *q*_*sp*_ as the flow of the export (in US dollars) of the product *p* by the state *s* (see the section *Database* for the data origin), the RCA is defined as:

$$\begin{array}{c} RCA_{sp}=\frac{\frac{q_{sp}}{\sum_{p^{\prime }}q_{sp^{\prime }}}}{\frac{\sum_{s^{\prime }}q_{s^{\prime }p}}{\sum_{s^{\prime }p^{\prime }}q_{s^{\prime }p^{\prime }}}}. \end{array}$$ (1)

Therefore, it is the ratio between the share of the export of product *p* with respect to the total export of State *s* divided by the share of the export of product *p* with respect to the total Brazilian export.

From the calculation of the RCA for each state-product pair, we build the binary state-product matrix *M*_*sp*_. We consider the state *s* an exporter of a product *p*, if *RCA*_*sp*_ ≥ 1 and, consequently, we set *M*_*sp*_ = 1. On the contrary, if *RCA*_*sp*_ < 1, we set *M*_*sp*_ = 0.

An analogous criterion is used to define the World countries-products matrix *M*_*cp*_ (see the section *Database*). This binary matrix shows which country has a comparative advantage in a certain product with respect to the World average [29, 30].

### (Endogenous) Fitness

Recently, different studies have shown the economic relevance of the diversification of the export basket for the competitiveness of a country [4, 6]. The matrix *M* shows a substantial nested structure highlighted by a strong triangularity, which can be interpreted in the following way: each country approximately exports all the possible products it has the capabilities to produce [5].

Here, considering the geographic size of Brazil and its federal structure, we assume that the same concept is also valid to understand the development and growth of its states. In this framework, we apply the Fitness algorithm to the states-products matrix of elements *M*_*sp*_ above defined [5], a statistical approach based on non linear maps coupling Fitness of states and Complexity of Products, to compare Brazilian states. The (Endogenous) Fitness algorithm is defined by the following iterative equations [6]:

$$\begin{array}{ccc} \{\begin{array}{c} {\tilde{F}}_{s}^{\left(n\right)}=\Sigma_{p}M_{sp}Q_{p}^{B\;\;\left(n-1\right)}\\ {\tilde{Q}}_{p}^{B\;\;\left(n\right)}=\frac{1}{\sum_{s}M_{sp}\frac{1}{F_{s}^{\left(n-1\right)}}} \end{array} & \to & \{\begin{array}{c} F_{s}^{\left(n\right)}=\frac{{\tilde{F}}_{s}^{\left(n\right)}}{{\langle {\tilde{F}}_{s}^{\left(n\right)}\rangle }_{s}}\\ Q_{p}^{B\;\;\left(n\right)}=\frac{{\tilde{Q}}_{p}^{B\;\;\left(n\right)}}{{\langle {\tilde{Q}}_{p}^{B\;\;\left(n\right)}\rangle }_{p}}. \end{array} \end{array}$$ (2)

The elements *M*_*sp*_ are the elements of the previously discussed binary states-products matrix. ${\tilde{F}}_{s}^{\left(n\right)}$ and ${\tilde{Q}}_{s}^{B\;(n)}$ are intermediate variables which are subsequently normalized at each iteration. The initial conditions satisfy the relations: ${\tilde{F}}_{s}^{\left(0\right)}=C$ and ${\tilde{Q}}_{p}^{B\;(0)}=C$, where we assume *C* = 1 for each state *s* and for each product *p* [6].

At each iteration of the algorithm, the Fitness of each state is proportional to the sum of its exported products weighted by their Complexity stressing the importance of having at the same time both a diversified export basket and the most complex possible products in it. The formula for the Complexity of a product is motivated by the following argument: the more the exporters of a product and the smaller their Fitness, the less its expected from the Complexity. In this manner, a state with low Fitness abruptly influences the Complexity of all the products it exports [6]. Therefore, an highly Complex product is made only by few countries/states with high Fitness, while a little Complex product can be made by all the countries/states, both with high and low Fitness. The stability and robustness of the algorithm has been studied in [6, 31] and the Fitness ranking of the states and the Complexity ranking of the products is unambiguously defined after a large enough number of iterations.

**Fig 1:**
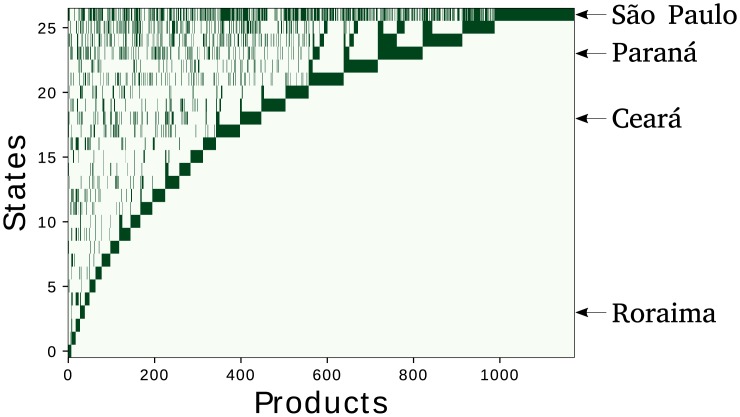
The binary matrix *M*_*sp*_ of the year 2015. Each row of the matrix represents a Brazilian state. States are ordered in terms of their Fitness from the smallest value (row 0) to the largest one (row 26). Analogously columns represent Products ordered in terms of their Complexity from the smallest value (column 0) to the largest one (column 1172). The matrix elements *M*_*sp*_ are drawn in dark green and the others in white. In the figure we highlight high Fitness states such as São Paulo and Paraná, a middle rank State such as Ceará and a low Fitness state such as Roraima.

Fig 1 shows the matrix *M*_*sp*_ of the year 2015, by ordering the states according to the Fitness (the upper the higher complexity), and the products according to the Complexity (the more right the higher the complexity).

**Fig 2:**
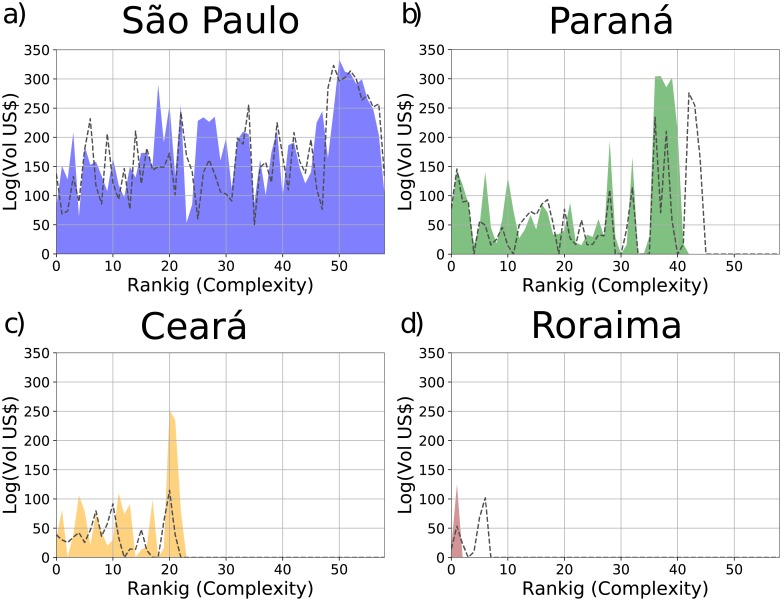
Products spectroscopy of the years 2005 (dotted lines) and 2015 (filled colors) of the states: a) São Paulo, b) Paraná, c) Ceará, and d) Roraima. The figures show the export volume (in US Dollars) of those states for each product with *M*_*cp*_ = 1 ordered according to their Complexity. Products are grouped in bins of 10 and the export volume in each bin are summed up.

In Fig 2 we show the products *spectroscopy* [32] of the years 2005 (dashed lines) and 2015 (filled colors) for few Brazilian states such as: São Paulo, Paraná, Ceará, and Roraima. The spectroscopy is a graphic representation of the export volume (in US Dollars) of a state for each product with *M*_*sp*_ = 1 ordered at increasing Complexity from left to right [32]. We subsequently group the products (10 for bin) and we summed the export volumes of each product inside each bin. The spectroscopy allows to compare the diversification and the Complexity of the exportation of the states. The figure shows the spectroscopy of high Fitness states such as São Paulo (diversified all along the Complexity spectrum) and Paraná (with a clear peak on medium-high Complexity products), a middle rank state such as Ceará and a low Fitness state such as Roraima (with few low Complexity exports). From the figure, it emerges that a very developed state such as São Paulo has a high flow of exports for a very diversified number of products with a bias towards the high Complexity ones. Paraná has a high peak in several complex products, while Roraima has only one peak in the less complex products. Ceará is a middle ground between the two.

### Exogenous Fitness

Here, we define the new Exogenous Fitness algorithm, an innovative method to calculate the Fitness of subnational entities of a country grounded on the measure of the products Complexity from the World-wide trade network. Exogenous Fitness is a coherent extension of the “classical” Fitness algorithm [6], with the assumption of an obvious concept: products have an intrinsic Complexity, reflected by the trade on the global World scale by all countries, while the trade from the regions of a single country may not represent well such intrinsic Complexity as it can be affected by local biases. In particular if we consider only Brazil to define the Complexity of the exported products, we can introduce local economic biases in its measure related to the peculiar features of Brazil economy. Indeed, as shown in Fig 1, there is a big range of products made only by few states that make the measure of Complexity very inaccurate. From this observation, it is natural to use as the best measure of Complexity of products the ones $Q_{p}^{W}$ extracted from the Fitness algorithm applied to the trade of goods of all World countries, i.e. we take:

$$\begin{array}{c} Q_{p}^{W}\equiv Q_{p}^{B}\equiv Q_{p}. \end{array}$$ (3)

Indeed, the Complexities of the products obtained applying the Endogenous Fitness to the World countries ($Q_{P}^{W}$) can be considered the same of the Complexities of the products inside Brazil ($Q_{P}^{B}$) and, therefore, we simply define them as *Q*_*p*_.

Therefore, the algorithm consists of two steps:

1. We apply the (Endogenous) Fitness (Eq 2) to the World countries, as previously done in [5–7]. The criterion adopted to determine if a country *c* is a “good” exporter of a given product *p* is again based on the RCA extended to all World countries: we set *M*_*cp*_ = 1 if *RCA*_*cp*_ ≥ 1 and *M*_*cp*_ = 0 otherwise (see the section *Database* for the source of the data). Applying the (Endogenous) Fitness algorithm to the matrix *M*_*cp*_, after a sufficiently large number of iterations the algorithm converges to the fixed point so that, we obtain the respective Fitness *F*_*c*_ for each country and the Complexity $Q_{p}^{W}$ for each product.
2. From the assumption Eq 3, we use as Complexity of the products exported by Brazilian states *Q*_*p*_ the values obtained by the Fitness algorithm applied to the export of all World countries. Therefore, we use the information in the matrix *M*_*sp*_ and the product Complexity *Q*_*p*_ to calculate the Fitness of the Brazilian states through the following formula:
  $$\{\begin{array}{c} {\tilde{F}}_{s}=\Sigma_{p}M_{sp}Q_{p}\\ F_{s}=\frac{{\tilde{F}}_{s}}{{\langle {\tilde{F}}_{s}\rangle }_{s}}. \end{array}$$ (4)

The relevance of developing the Exogenous Fitness measure is two folds. First of all, using world wide data we extract all the information to compute the Complexity of products to better compute the Fitness of states. Since the algorithm works by exploiting differences of capabilities, using world wide data we gain additional information related to the export baskets of countries with a wider range of Fitness and capabilities. Of course we still expect the two measures to be highly correlated in rank, in particular for a country like Brazil that contains such a vast array of development levels. As we will see in section *Comparison with other techniques*, this is indeed the case. The second reason is that the Exogenous Fitness allows to have for states Fitness values comparable with those of countries. Indeed, while the ranking between Exogenous and Endogenous Fitness are highly correlated, their actual values and distributions are vastly different. As detailed explained in the paper [33], while the ranking for the Fitness measure is always well defined, the shape of the matrix directly affects the convergence properties of the algorithm to a polarized distribution. Employing the Exogenous Fitness method we have smoothly changing values that allows for the forecasting exercises of section *Results*.

## Overview of Brazil

**Fig 3:**
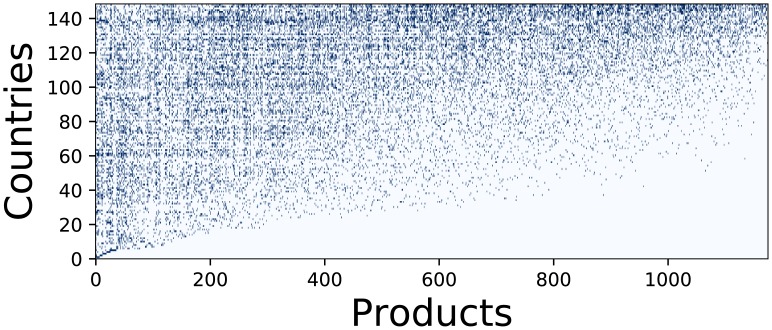
The binary matrix *M*_*cp*_ of the year 2015. The rows of the matrix represent the World countries ordered according to their Fitness with row 0 for the country with the lowest Fitness and row 147 for the one with the highest Fitness. Analogously columns represent Products ordered in terms of their Complexity from the lowest one at column 0 to the highest one at column 1174. The elements *M*_*cp*_ = 1 are represented as blue dots.

First, we analyze Brazil as a whole applying the (Endogenous) Fitness to World countries in the time interval from 1995 to 2015. In Fig 3 we show the matrix *M*_*cp*_ of the World countries of year 2015 obtained by ordering the countries according to the Fitness and the products according to the Complexity. In that year, Brazil is ranked in the 44th/147 position (equivalent to the raw 103 in Fig 3).

**Fig 4:**
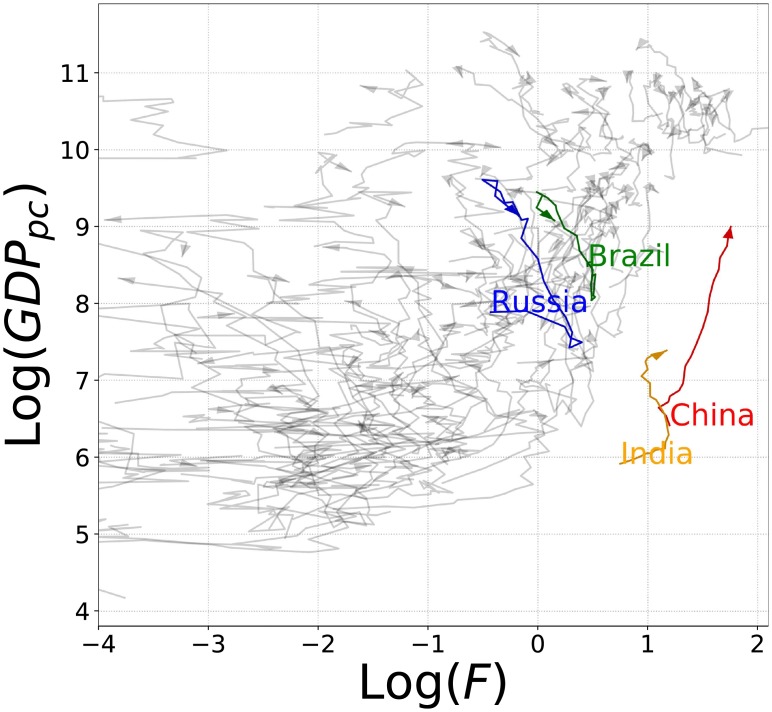
Dynamics of the World countries in the Fitness-Income plane. The figure shows the dynamics (from the year 1995 to the year 2015) of World countries in the Fitness-Income plane in logarithmic scale. We emphasize the BRIC countries: Brazil in green, Russia in blue, China in red, and India in orange.

In Fig 4, we show the dynamics of the World countries in the Fitness-Income plane emphasizing the BRIC countries (Brazil in green, Russia in blue, India in orange, and China in red). The figure shows that India and China have in 1995 lower values of GDP_*p*_ than Brazil and Russia, but higher values of Fitness. According with [7], this difference justifies the dynamics in the plane of the four countries for the next years. Indeed, India and China continued their economic growth during the following years, while Russia and Brazil entered a period of recession [34].

In order to zoom on the differences among the dynamics of the BRIC countries, we analyze the variation of the Fitness of such countries during the interval from 2003 and 2013. The variation of the Fitness can have two different causes: (i) changes in the export basket, (ii) changes in the products Complexity. We can decompose the variation of Fitness [35] as:

$$\begin{array}{l} \Delta {\tilde{F}}_{c}={\tilde{F}}_{c}(t_{1})-{\tilde{F}}_{c}(t_{0})=\sum_{p}M_{cp}(t_{1})Q_{p}(t_{1})-\sum_{p}M_{cp}(t_{0})Q_{p}(t_{0})=\\ =\sum_{p}\Delta M_{cp}\frac{Q_{p}(t_{1})+Q_{p}(t_{0})}{2}+\sum_{p}\Delta Q_{p}\frac{M_{cp}(t_{1})+M_{cp}(t_{0})}{2}. \end{array}$$ (5)

where we have indicated with Δ*X* = *X*(*t*_1_) − *X*(*t*_0_) for a generic quantity *X*. The first term in the last step of the equation is the contribution to $\Delta {\tilde{F}}_{c}$ due to the variation in the export basket, while the second one is the term due to variation of products Complexities. In Table 1, we show both the percentage variations due to the two terms. The results show a deep decrease of both terms for Russia and we can see how the loss of competitiveness of Brazil is mostly due to the drop of products that were previously exported, and not so much related to the change in complexity of those products. In contrast China has increased its export basket and the Complexity of the exported products. Instead, India in 2013 exports more complex products, but has decreased its exports diversification.

**Table 1:** Fitness variation from 2003 to 2013 of BRIC countries.

	Variation due to changes in the export basket	Variation due to changes in the products Complexities
Brazil	-43%	-6%
Russia	-37%	-21%
China	+32%	+18%
India	-18%	+2%

**Fig 5:**
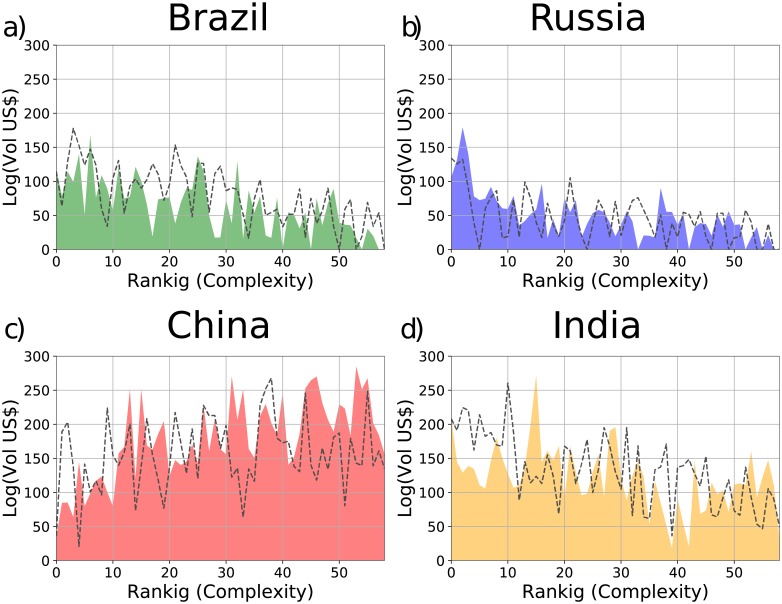
Products spectroscopy of the years 2005 (dotted lines) and 2015 (filled colors) of the countries: a) Brazil, b) Russia, c) China, and d) India. The figures show the export volume (in US Dollars) of those states for each product with *M*_*cp*_ = 1 ordered in terms of their Complexity. The products have been grouped (10 for bin) and the export volumes of each product inside each bin have been summed.

Furthermore, we show in Fig 5 the products spectroscopy [32] for the BRIC countries of the year 2005 (dotted lines) and 2015 (filled colors). The figure shows that Brazil and Russia have a high exportation only of simple products, while India and China have a high exportation of complex products.

Therefore, Figs 4 and 5 and Table 1, show that China and India both have a diversified export basket and export complex products. Such factors determine a high Fitness and consequently a growth of the GDP_*p*_ in the subsequent years. On the contrary Brazil and Russia export simple products with a consequently low Fitness so that these countries entered a recession period [34].

In the next section we show the results of a deepened analysis of the internal economy of Brazil through the application of the Exogenous Fitness to the Brazilian states.

## Results

**Fig 6:**
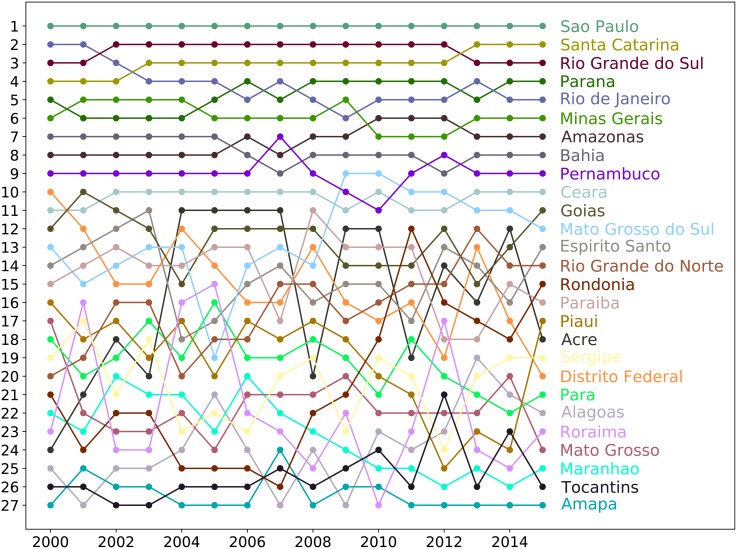
Time evolution of the ranking of Brazilian states according to the Exogenous Fitness algorithm. The figure shows the time evolution of the ranking of the Brazilian states according to the Fitness obtained through the Exogenous Fitness algorithm applied to the time interval 2000-2015.

We applied the Exogenous Fitness algorithm to the Brazilian states in the time interval from 2000 to 2015 obtaining for each year both well-defined values of Fitness for each Brazilian state, and the ranking of states in terms of their Fitness (shown in Fig 6).

**Fig 7:**
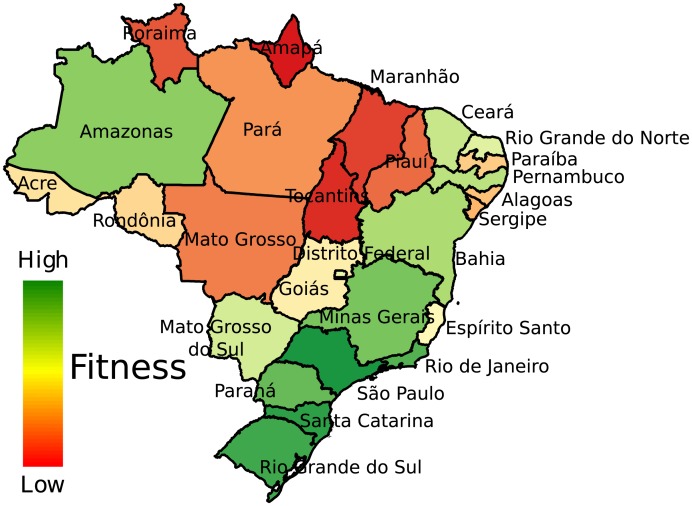
Fitness map of the Brazilian states. The colors in the map vary from green (high Fitness) to red (low Fitness) and they show the differences of the Fitness among the Brazilian states.

We show in Fig 7 a map of Brazil where each state is colored according to its Fitness. From the figure, it emerges Southern states have larger Fitness, and therefore have a better economic development, than Northern states. This result is in agreement with other monetary and non-monetary indices such as the Human Development Index (HDI) and the GDP [27].

Furthermore, we show in Table 2 the variation from 2003 to 2013 of the Fitness ($\Delta \tilde{F_{s}}$) for several states such as: São Paulo (1st in Fitness ranking of year 2013), Paraná (5th in Fitness ranking of year 2013), Ceará (10th in Fitness ranking of year 2013), and Roraima (24th in Fitness ranking of year 2013). From both Fig 2 and Table 2 we observe that São Paulo has a diversified export basket with high peaks in complex products and, at the same time, it increases both the export basket and the Complexity of the exported products in the considered time period. Paraná and Ceará, in contrast with the aggregate behavior of Brazil, in the same period grew in diversification becoming more competitive—even in the face of a minor decline in the complexity of their exported products. Roraima, on the contrarty, shows a deep decrease in the diversification.

**Table 2:** Fitness variation from 2003 to 2013 of the states: São Paulo, Paraná, Ceará, and Roraima.

	Variation due to changes in the export basket	Variation due to changes in the products Complexities
São Paulo	+2%	+2%
Paraná	+59%	-7%
Ceará	+53%	-10%
Roraima	-37%	+6%

As mentioned in the previous section, Fig 4 presents the dynamics of World countries in the Fitness-GDP_*p*_ plane. It shows a high degree of heterogeneity of the dynamics of countries. Indeed, the plane can roughly be divided into two regions: one with an unpredictable “chaotic” regime of the evolution of countries, and the other with a predictable “laminar” regime. In order to overcome the limitations of linear regressions, Cristelli *et al* [7] proposed an innovative data-driven non-parametric prediction scheme called the *Selective Predictability Scheme* (SPS). It is inspired by the so-called *method of analogues* [36, 37] and through a *measure of concentration* it delimits predictability regions inside the Fitness-Income plane. The measure of concentration consists in dividing the plane into a grid and analyzing the time evolution of the distribution of countries inside each box with at least five countries inside.

**Fig 8:**
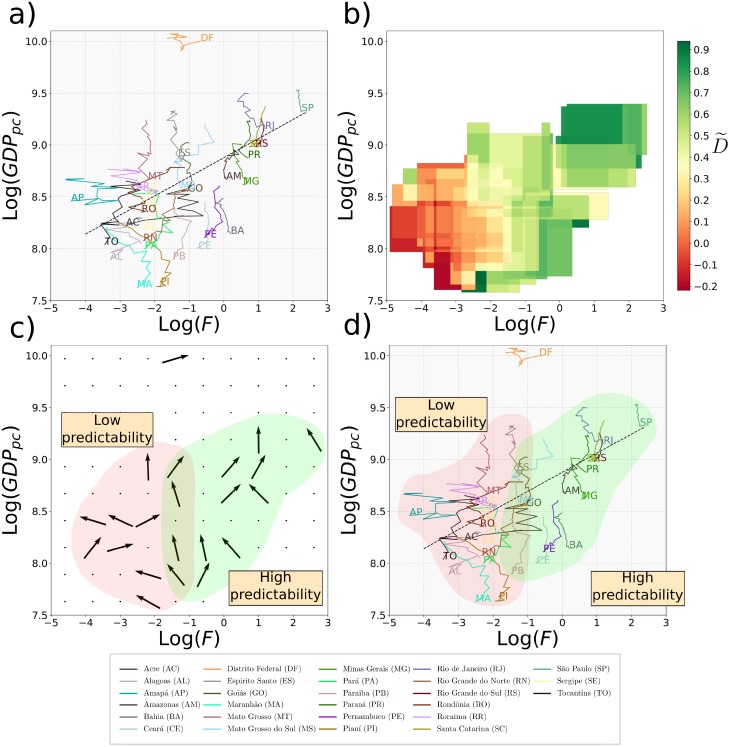
Dynamics of Brazilian states in the Fitness-Income plane. *a*) The figure shows the evolution (from 2000 to 2015) of the Brazilian states in the Fitness-Income plane in logarithmic scale. The dotted black line in the figure shows the expected level of GDP_*p*_ given the level of Fitness and it is the result of the minimization of the Euclidean distance of the states from the line, weighted by the states GDP. *b*) The figure shows the coefficient $\tilde{D}$ calculated considering a time window from 2003 to 2013. The color varies from green (where the versors of evolution tend to be parallel), to red (where the versors tend to be unevenly directed). *c*) The figure shows a grid where for each cell we calculate the versor of the sum vector. From the figure two regions appear: the first one where the versors tend to be parallel in the direction of a high GDP_*p*_ (shown in green); and the second one where the versors tend to be unevenly directed (shown in red). Fig 8*b* and *c* together show that there is a region (green) of high predictability of motion in direction of a high GDP_*p*_; and a region (red) of low predictability of motion. *d*) The figure shows the dynamics (from 2000 to 2015) of the Brazilian states in the Fitness-Income plane highlighting in green the states in the high predictability region and in red the states in the low predictability one.

In analogy with what has just been explained for World countries, in Fig 8a, we show the time evolution of the real GDP_*p*_ as a function of the Fitness (obtained implementing the Exogenous Fitness algorithm), for each Brazilian state in the period 2000-2015. The dotted black line in the figure shows the expected level of GDP_*p*_ given the level of Fitness and it is the result of the minimization of the Euclidean distance of the states from the line, weighted by the state GDP. From the figure emerges an heterogeneous dynamics similar to the dynamics of World countries that cannot be analyzed through a linear regression. Also the *measure of concentration* is not appropriate in this case. Indeed the reduced number of Brazilian states (27) compared with the number of World countries (146) makes this measure inappropriate for the internal analysis of Brazil. In order to have a significant number of cells with at least five states, the granularity of the grid should be too broad to analyze the evolution of the distribution.

Therefore, in order to validate the predictability of the dynamics of the states in the Fitness-Income plane, here we develop a novel intuitive method, the *measure of direction*. First of all let us fix the time window [*t*_1_, *t*_2_] in which we want to study the evolution of each state in the plane log(*Fitness*) − log(*GDP*_*p*_). The time lag Δ = *t*_2_ − *t*_1_ has to be taken large enough to get a sufficient noise reduction in the dynamics. We choose *t*_1_ = 2003 and *t*_2_ = 2013. Second, we divide the plane in a fine grid of 100 × 100 cells and we define two bandwidth; one for the x-axis, and the other for the y-axis. For each cell, we define around its centroid a threshold area of sides given by the two bandwidths. Then, for each cell *k* with at least three states at the time *t*_1_ inside its threshold area, we computed the average dot product $\tilde{D_{k}}$:

$$\begin{array}{c} {\tilde{D}}_{k}=\frac{2}{N(N-1)}\sum_{i<j}^{1,N}{\hat{v}}_{i}\cdot {\hat{v}}_{j}, \end{array}$$ (6)

where ${\hat{v}}_{i}=\frac{{\vec{v}}_{i}}{v_{i}}$ where ${\vec{v}}_{i}=\left[log\left(F_{i}\left(t_{2}\right)\right)-log\left(F_{i}\left(t_{1}\right)\right)\right]\hat{i}+\left[log\left(GDP_{p{}_{i}}\left(t_{2}\right)\right)-log\left(GDP_{p{}_{i}}\left(t_{1}\right)\right)\right]\hat{j}$ and $\hat{i}$ and $\hat{j}$ are respectively the versors in the Fitness and GDP_*p*_ directions. *N* is the number of states with starting point inside the threshold area of cell *k*. The coefficient $\tilde{D_{k}}$ gives the average cosine among the versors of all states initially inside the threshold area of cell *k* and varies from (−1, 1]. It measures the dispersion of the directions of evolution in the plane in the time window [*t*_1_, *t*_2_] of all states initially in the threshold area of cell *k*: when it is close to 1 all states initially in the threshold area of cell evolve in a coherent parallel way. The smaller is ${\hat{D}}_{k}$ the larger the dispersion of these trajectories. A color map of the coefficient $\tilde{D}$ in the different cells is shown in Fig 8b. From the figure it emerges that there is a region where the directions of evolution of the states tend to be parallel (showed in green) and a region where the directions of motion tend to be unevenly directed (showed in red). Increasing/decreasing the bandwidths and, therefore, the threshold area only changes the resolution of the image, but the two regions remain well-defined. In Fig 8b we used an x-axis bandwidth 0.86, and a y-axis bandwidth 0.38, providing an almost continuous variation of the colors map.

In order to investigate which is the main direction of the versors in the green region and the further directions in the red region, we divided the plane into a broader grid (10x10). For each cell we sum all the vectors inside it and then we calculate the versor of the sum vector. We show the result in Fig 8c. From one hand, from the figure we can observe a region where the states tend to evolve in the same direction (shown in green). Therefore, in this region, the future evolution of countries is predictable with good confidence. On the other hand, another region (shown in red) can be detected where the versors tend to be unevenly directed. The dynamics of the states in this region is basically unpredictable. Furthermore, in the middle of the two, there is a region of transition, shown in the figure by the overlapping of the two colors.

Lastly, in Fig 8d we show the dynamics of the states in the Fitness-Income plane highlighting in green the states with high predictability of the motion and in red those with low predictability. From the figure emerges that states as Ceará, Pernambuco, and Bahia, despite having low values of GDP, are in a region of high Predictability and, therefore, they will probably continue to growth in the same direction. While for states as Acre, Tocantins, or Alagoas the dynamics is more chaotic and predictions are less reliable.

## Comparison with other techniques

In this section we compare the results obtained implementing the Exogenous Fitness with the results of the Endogenous Fitness and the ones published by Dataviva [27] obtained by applying the Economic Complexity Index (ECI).

### Exogenous Fitness and Endogenous Fitness

**Fig 9:**
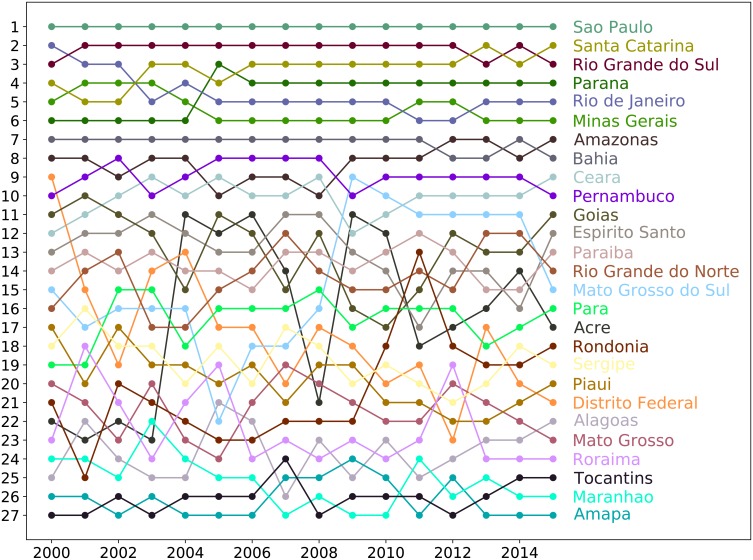
Time evolution of the ranking of Brazilian states according to the (Endogenous) Fitness algorithm. The figure shows the time evolution of the ranking of the Brazilian states in terms of the Fitness obtained through the (Endogenous) Fitness algorithm applied during the time interval 2000-2015.

We apply the (Endogenous) Fitness algorithm to the Brazilian states in the time interval from 2000 to 2015 obtaining the time evolution of the ranking of the states according to such kind of Fitness (shown in Fig 9). Calculating the Spearman correlation coefficient between the ranking obtained through the Exogenous and the Endogenous Fitness for each year in the analyzed time interval, we obtain an average value ${\tilde{\rho }}_{ExEn}=0.97$. This result shows a strong correlation between the rankings obtained through the two different Fitness algorithms.

The Endogenous Fitness algorithm provide us a well-defined annual ranking of the Brazilian states, but not well-defined quantitative values of Fitness and products Complexity. In fact, all Fitness values except one tend to zero. After a fairly high number of iterations, however, the ranking of states stabilizes, and there are no more changes of ranking among the states. This circumstance is already been studied [33] and it is due to the shape of the matrices *M*_*sp*_. Indeed the *external area* (where *M*_*sp*_ = 0) is greater than the *internal area* (where almost all elements *M*_*sp*_ = 1) for each analyzed year.

### Exogenous Fitness and ECI

**Fig 10:**
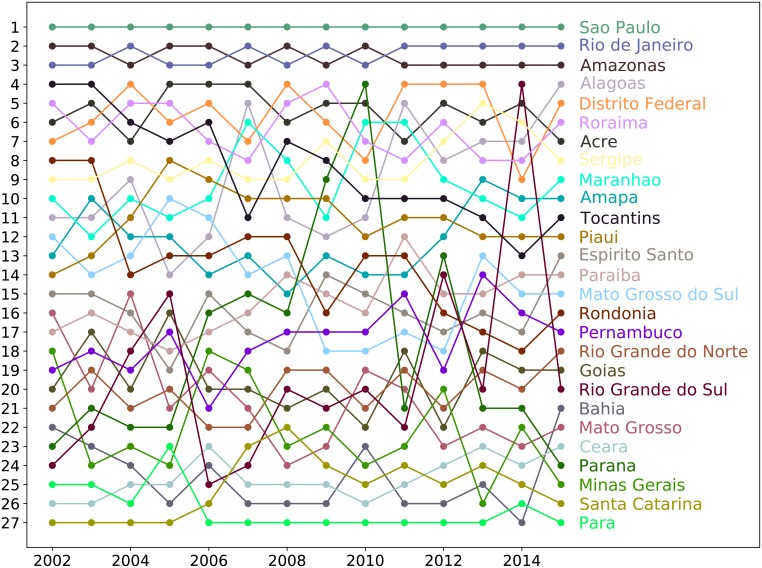
Time evolution of the ranking of Brazilian states according to the ECI algorithm. The figure shows the time evolution of the ranking of the Brazilian states during the period 2002-2015 in terms of the ECI, directly downloaded by the Dataviva platform [27].

In Fig 10 we show the time evolution (from 2002 to 2015) of the ranking of the Brazilian states according to ECI, directly downloaded by the Dataviva platform [27]. Therefore, in order to compare the ranking obtained through the Exogenous Fitness algorithm and the ECI algorithm, we calculate the annual Spearman correlation coefficient between the two rankings in the period 2002-2015, obtaining an average value ${\tilde{\rho }}_{ExECI}=-0.14$. This result shows an almost total absence of correlations between the two rankings, i.e. between the two algorithms.

Indeed, already from a qualitative point of view, ECI ranking seems to be unrealistic. For example, it ranks rich states in GDP, but also with high HDI [27], such as Santa Catarina or Paraná, in the last positions (respectively 26th and 24th position in 2015). Moreover, the state of Alagoas (last in HDI ranking of 2014 [27]) is unrealistically ranked in 4th position in the 2015.

**Fig 11:**
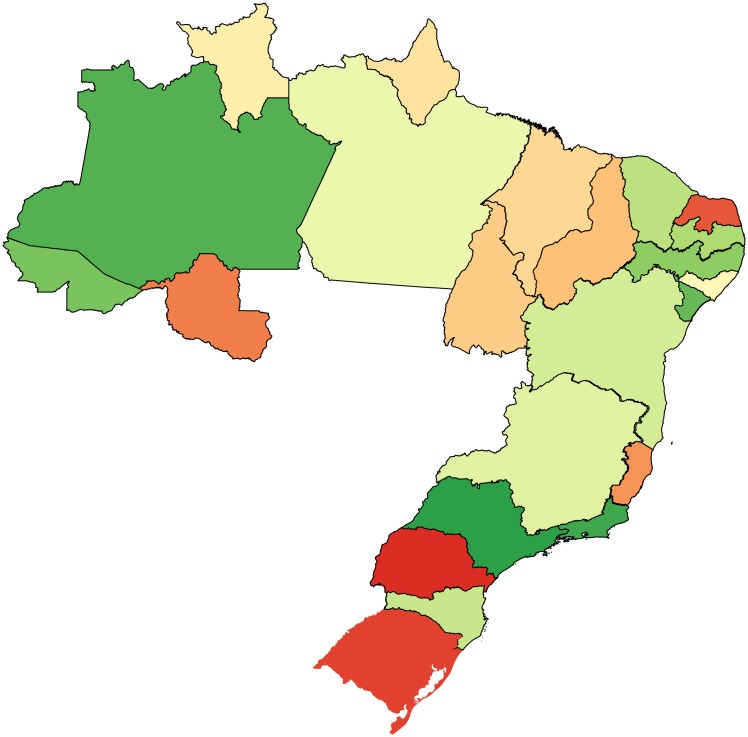
ECI map of the Brazilian states. The colors in the map vary from green (high ECI) to red (low ECI) and they show the variation of the ECI across the Brazilian states.

In Fig 11, we show the map of Brazil where each state is colored according to its ECI. From the figure, it emerges that there is no geographic coherence among the ECI of the different states. For instance the figure shows that the state of Santa Catarina has a high ECI, but it is in the middle between the states of Rio Grande do Sul and Paraná that have a low ECI.

**Fig 12:**
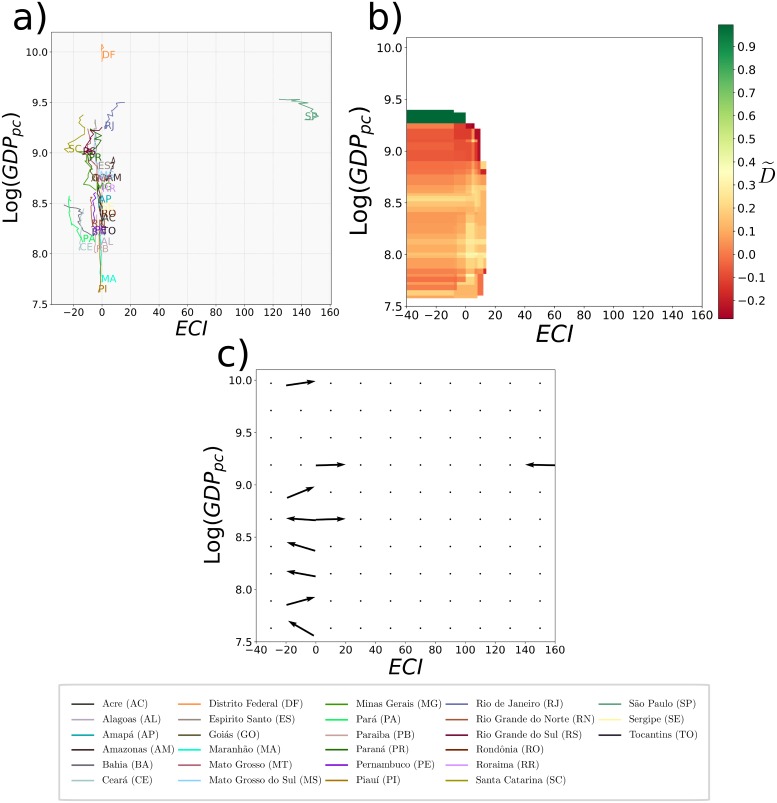
Evolution of Brazilian states in the ECI-Income plane. *a*) The figure shows the dynamics (from 2002 to 2015) of the Brazilian states in the ECI-Income plane, where the GDP_*p*_ is in logarithmic scale. Only the state of São Paulo and the Distrito Federal appear to be clearly distinguishable from the rest of the states. All the others states are indeed concentrated in a small region of the graph. *b*) The figure shows the coefficient $\tilde{D}$ calculated considering the time interval 2003-2013. Colors vary from green (where the versors tend to be parallel), to red (where the versors tend to be unevenly directed). From the figure we can therefore verify that there is a low predictability of the evolution of all the states. *c*) Here we show a grid where for each cell we calculate the versor of the sum vector. From the figure we see that there is no privileged direction, indeed the vectors are unevenly directed.

Furthermore, we show in Fig 12a the evolution of Brazilian states in the ECI-Income plane, where the income is in logarithmic scale. In Fig 12b, we show the coefficient $\tilde{D}$ above defined but applied to ECI instead to *log*(*Fitness*) and in Fig 12c the directions of motions. Differently from the results obtained through the application of the Exogenous Fitness (Fig 8), using the ECI index the dynamics of the states is unpredictable. Indeed, all the states except São Paulo and the Distrito Federal are concentrated in a small region of the plane and, therefore, totally indistinguishable.

## Discussion

In this paper we first compared the dynamics of Brazil in the Fitness-Income plane with the other BRIC countries. In Fig 4, we observed that IC (India and China) countries, both with a high Fitness compared to the BR (Brazil and Russia) countries, grow in GDP_*p*_ for the entire analyzed time interval. Table 1 shows that IC improve the Complexity of export baskets in the analyzed time interval, and China even shows an improvement of the diversification. Instead, BR countries did not invested in diversification and in Complexity of the exported products (as shown in Table 1). These results strengthen an hypothesis previously formulated in [7]: Fitness is the driving force behind growth.

In the second part of the paper, we introduced a new algorithm called “Exogenous Fitness” to calculate the Fitness of subnational entities and we applied it to the states of Brazil. The comparison between the Fitness and the GDP_*p*_ showed an heterogeneous dynamics of the Brazilian states in the Fitness-Income plane. Indeed, two regions are distinguishable in the plane: one with high predictability and the other with low predictability. Here, we have shown that economic forecasting is possible for those states in the high predictability region, while it is not for those in the low predictability region. As a consequence of this analysis Fitness seems to be the driving force behind growth. Indeed, the dynamics in the high predictability region is characterized by high values of Fitness, while high value of GDP_*p*_ is not a good signature of growth. The heterogeneous dynamics observed for the Brazilian states shows a strict analogy with the heterogeneous dynamics observed for the World countries [7]. Furthermore, by comparing the export “spectroscopy” of BRIC countries with the one of Brazilian states of São Paulo, Paraná, Ceará, and Roraima, and, comparing the variations of the Fitness, we observe that countries/states with diversified export baskets produce high complex products and grew in GDP_*p*_ in the considered period. This observation can be important for the evaluation of perspectives of economic growth for Brazilian states, and, more generally, for developing countries.

The time evolution of the ranking obtained through the Exogenous Fitness algorithm shows that developed states in the top part of the ranking change little their positions, with a smooth slow motion. On the contrary states in the inferior part of the ranking changes drastically their position during the analyzed time-interval. These facts are probably due to the stability of the developed states that are in the high predictability region of the Fitness-GDP_*p*_ plane and the instability of the states in the low predictability region.

Finally, we showed the non-correlation (${\tilde{\rho }}_{ExECI}=-0.14$) between the ranking obtained though the Exogenous Fitness algorithm and the results of the ECI published by Dataviva [27]. Analyzing qualitatively the ranking of the states according to ECI, we argued that this ranking appears quite unrealistic. Therefore, we propose here the Exogenous Fitness algorithm as its valid substitute. Instead, comparing the Exogenous and (Endogenous) Fitness we obtained a strong correlation (${\tilde{\rho }}_{ExEn}=0.97$) for what concerns the ranking of states. This result shows that the two algorithmic tools are almost similar in identifying the ranking of the states, but just the Exogenous Fitness algorithm provides also stable quantitative values of the Fitness, in addition to the ranking.

## Appendix A

### Dataset

The vast majority of data used in this paper is published by *DataViva* [27]. It is an open access platform that easily allows the access to a large amount of Brazilian socioeconomic data. The database is provided by the Brazilian *Ministries*: of *Employment* (MTPS), *Development*, *Industry and International Trade (MDIC)* and *Education* (MEC). The project is an initiative of the *Government of the state of Minas Gerais*, *Minas Gerais Investment*, *Trade Promotion Agency* (INDI) and the *Fundação de Amparo à Pesquisa do Estado de Minas Gerais* (FAPEMIG) [27, 38] in collaboration with the *Sistema Mineiro de Inovaçao* (SIMI) [39], *Big Data Corp* [40] and the *MIT Media Lab* [41]. The first version was published in November 2013 and the last one, the 3.0 version, in May 2015.

The platform includes data about imports/exports products, trade partners, occupation, economic activities, basic education, higher education and universities. All data are available in several levels of aggregation: region, state, mesoregion, microregion and municipality. The crossover among data and level of aggregations allows users to access more than 1 billion visualizations.

The visualization is made through some graph types, such as: Tree Map, Stacked, Geo Map, Network, Rings, Scatter, Compare, Occugrid, Line, Box Plot and Bar Chart. Furthermore, each data and aggregations is downloadable, and easily accessible through the API architecture [42].

Here, we use the export data of each Brazilian state for the entire time interval from 2000 to 2015. Furthermore, *DataViva* provides the data of total GDP and the total population for each state for the same time interval. Combining these with the GDP deflator *GDP*_*defl*_, published by the *World Bank* [43], we find the real GDP *per capita* of each state as:

$$\begin{array}{c} GDP_{p}^{real}=\frac{1}{N}\frac{GDP}{GDP_{defl}}100, \end{array}$$ (7)

where *N* is the total population of each state.

Concerning World export data, used to define the matrix *M*_*cp*_ of the World countries and to calculate the products complexity, we use data from BACI dataset [29] that is grounded on the COMTRADE dataset [30]. The database, in its extension, contains data about more than 200 countries and 5000 products classified according to a 4 digit code with categorization *Harmonized System* 2007 [44]. Data are extracted from the year 2000 to 2015. The time evolution of the GDP *per capita* of each country is published by *World Bank* [45].
