# Correction: Dynamics in the Fitness-Income plane: Brazilian states vs World countries

**DOI:** 10.1371/journal.pone.0217034

**Published:** 2019-05-15

**Authors:** Felipe G. Operti, Emanuele Pugliese, José S. Andrade, Luciano Pietronero, Andrea Gabrielli

Fig 2 “Products spectroscopy of the years 2005 (dotted lines) and 2015 (filled colors) of the states: a) São Paulo, b) Paraná, c) Ceará, and d) Roraima.” is incorrect. Please see the correct Fig 2 here.

**Fig 2:**
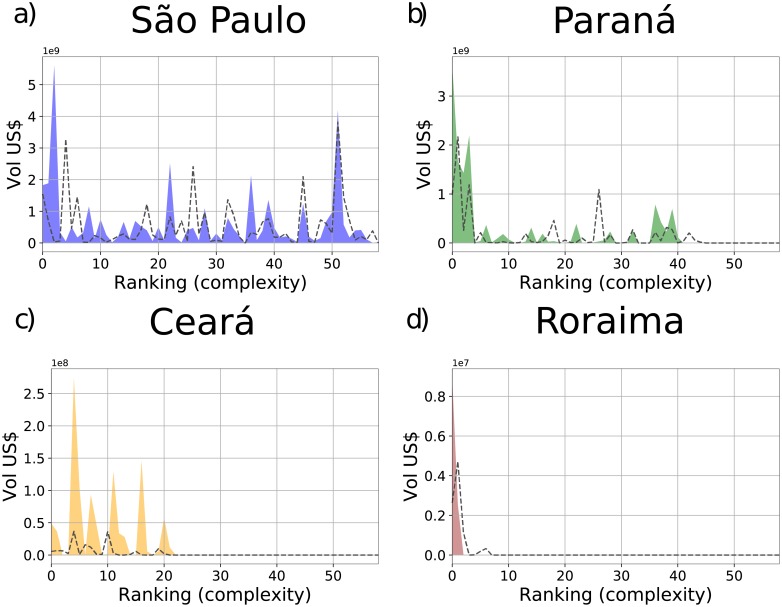
Products spectroscopy of the years 2005 (dotted lines) and 2015 (filled colors) of the states: a) São Paulo, b) Paraná, c) Ceará, and d) Roraima. The figures show the export volume (in US Dollars) of those states for each product with *M*_*cp*_ = 1 ordered according to their Complexity. Products are grouped in bins of 10 and the export volume in each bin are summed up.

Fig 5 “Products spectroscopy of the years 2005 (dotted lines) and 2015 (filled colors) of the countries: a) Brazil, b) Russia, c) China, and d) India.” is incorrect. Please see the correct Fig 5 here.

**Fig 5:**
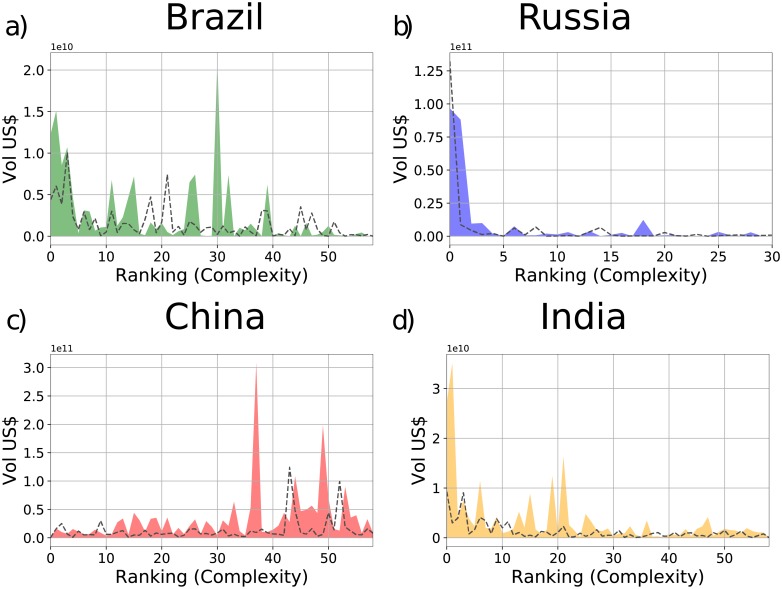
Products spectroscopy of the years 2005 (dotted lines) and 2015 (filled colors) of the countries: a) Brazil, b) Russia, c) China, and d) India. The figures show the export volume (in US Dollars) of those states for each product with *M*_*cp*_ = 1 ordered in terms of their Complexity. The products have been grouped (10 for bin) and the export volumes of each product inside each bin have been summed.

Fig 11 “ECI map of the Brazilian states.” is incorrect. Please see the correct Fig 11 here.

**Fig 11:**
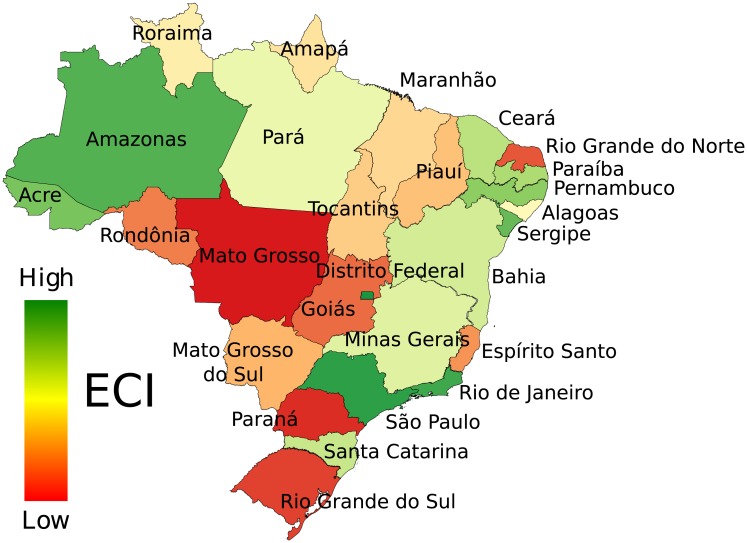
ECI map of the Brazilian states. The colors in the map vary from green (high ECI) to red (low ECI) and they show the variation of the ECI across the Brazilian states.
